# First Post-Operative Urinary Kidney Injury Biomarkers and Association with the Duration of AKI in the TRIBE-AKI Cohort

**DOI:** 10.1371/journal.pone.0161098

**Published:** 2016-08-18

**Authors:** Steven G. Coca, Girish N. Nadkarni, Amit X. Garg, Jay Koyner, Heather Thiessen-Philbrook, Eric McArthur, Michael G. Shlipak, Chirag R. Parikh, TRIBE-AKI Consortium

**Affiliations:** 1 Division of Nephrology, Department of Medicine, Icahn School of Medicine at Mount Sinai, New York, New York, United States of America; 2 Division of Nephrology, Department of Medicine, Western University, London, Ontario, Canada; 3 Section of Nephrology, Department of Medicine, University of Chicago, Pritzker School of Medicine, Chicago, Illinois, United States of America; 4 Division of General Internal Medicine, San Francisco VA Medical Center, University of California, San Francisco, California, United States of America; 5 Program of Applied Translational Research, Department of Internal Medicine, Yale University School of Medicine, New Haven, Connecticut, United States of America; Robert Bosch Krankenhaus, GERMANY

## Abstract

**Background:**

We previously demonstrated that assessment of the duration of AKI, in addition to magnitude of rise in creatinine alone, adds prognostic information for long-term survival. We evaluated whether post-operative kidney injury biomarkers in urine collected immediately after cardiac surgery associate with duration of serum creatinine elevation.

**Methods:**

We studied 1199 adults undergoing cardiac surgery in a prospective cohort study (TRIBE-AKI) and examined the association between the levels of five urinary biomarkers individually at 0–6 hours after surgery: interleukin-18 (IL-18), neutrophil gelatinase-associated lipocalin (NGAL), kidney injury molecule-1 (KIM-1), liver fatty acid binding protein (L-FABP) and albumin with duration of serum creatinine-based AKIN criteria for AKI (0 (no AKI), 1–2, 3–6, ≥7 days).

**Results:**

Overall, 407 (34%) patients had at least stage 1 AKI, of whom 251 (61.7%) had duration of 1–2 days, 118 (28.9%) had duration 3–6 days, and 38 (9.3%) had duration of ≥7 days. Higher concentrations of all biomarkers (per log increase) were independently associated with a greater odds of a longer duration of AKI; odds ratios and 95% confidence intervals using ordinal logistic regression were the following: IL-18: 1.22, 1.13–1.32; KIM-1: 1.36, 1.21–1.52; albumin 1.20, 1.09–1.32; L-FABP 1.11, 1.04–1.19; NGAL 1.06, 1.00–1.14). AKI duration of 7 days or longer was associated with a 5-fold adjusted risk of mortality at 3 years.

**Conclusions:**

There was an independent dose-response association between urinary levels of injury biomarkers immediately after cardiac surgery and longer duration of AKI. Duration of AKI was also associated with long term mortality. Future studies should explore the potential utility of these urinary kidney injury biomarkers to enrich enrollment of patients at risk for longer duration of AKI into trials of interventions to prevent or treat post-operative AKI.

## Introduction

After cardiac surgery, AKI is seen in up to 30% of patients and is associated with adverse outcomes including increased length of stay and mortality.[1][2] In addition to the magnitude of serum creatinine rise, duration of AKI has been shown to be independently associated with poorer long-term survival after cardiac surgery and non-cardiac surgery.[3][4] Recent advances have led to the discovery and validation of several novel biomarkers that have demonstrated utility in ascertaining AKI before rise in serum creatinine. [5] These include kidney injury biomarkers such as neutrophil gelatinase-associated lipocalin (NGAL), interleukin-18 (IL-18), kidney injury molecule-1 (KIM-1), and liver fatty acid binding protein (L-FABP). We have previously demonstrated that several of these biomarkers are independently associated with the development of AKI. [6] The expectation has been that biomarkers would assist in drug development to enrich event rates to improve efficiency and statistical power of clinical trials. Transient AKI or short duration AKI will dilute the ability to detect the effect of any new intervention, since even those in the non-intervention arm will have rapid recovery of renal function. Thus, the duration of AKI perhaps serves as a better indicator of AKI, not only for its association with poor outcomes, but to serve as an endpoint in AKI prevention or treatment trials.

In order to enrich a treatment trial for AKI, one could consider leveraging the immediate post-operative injury biomarkers to predict those patients at higher risk for longer duration of AKI. Since the relationship of injury biomarkers with the duration of AKI has not yet been elucidated, we sought to examine the association between biomarkers of kidney injury and duration of AKI in the TRIBE-AKI (Translational Research Investigating Biomarker Endpoints in Acute Kidney Injury) cohort. We were also interested in validating the association of duration of AKI on long term mortality in this cohort.

## Materials and Methods

### Patient Cohort

The TRIBE-AKI cohort is a prospective cohort study of patients undergoing cardiac surgery at high risk for postoperative AKI, enrolled at six academic medical centers in North America from July 2007 to December 2009. Patients were excluded if they experienced AKI before the surgery, had undergone kidney transplantation, had a history of ESRD, or a baseline serum creatinine of >4.5 mg/dl. All the patients provided written informed consent, and the respective institution’s review board approved the protocol. The detailed methods for sample collection have been described previously.[7] We included 1199 adults undergoing cardiac surgery in the TRIBE-AKI cohort that survived to discharge, to eliminate confounding by competing risk of early pre-operative death with AKI duration, and also to allow for assessment of duration of AKI for long-term mortality. AKI was defined by a rise in serum creatinine of at least 0.3 mg/dL or 50% from baseline to peak post-operative value at any time during the initial post-operative hospitalization. To obtain mortality data, we employed several overlapping approaches. For patients from the United States, we called patients’ homes, searched the National Death Index, and reviewed hospital records. For patients from Canada, we used phone calls, as well as data held at the ICES (Institute for Clinical Evaluative Sciences) to acquire vital status. These data sets were linked using unique, encoded identifiers and analyzed at ICES.

### Sample Collection and Biomarker Assays

We collected urine specimens preoperatively and daily upto 5 postoperative days. The first postoperative sample was collected 0–6 hours after surgery. For the first 24 hours postoperatively, urine samples were collected every 6 hours. The remaining daily blood and urine samples were obtained at the time of routine morning blood collection done for clinical care. Specimen collection was stopped on postoperative day 3 in subjects who had not yet had an increase in serum creatinine. We obtained fresh urine samples from urinary catheters and centrifuged the samples to remove cellular debris. We aliquoted urine supernatant into cryovials and stored the samples at −80°C until biomarker measurement. No additives were added.[7] The details regarding the biomarker assays have been described previously.[8]

### Statistical Methods

The distributions of the biomarkers were skewed, so we log transformed all biomarkers and analyzed them continuously and by quintiles. We compared continuous variables with one-way analysis of variance and categorical variables with chi-squared tests across categories of AKI duration. Violin plots were created to visually compare biomarker levels at 0–6 hours post-operatively across these AKI categories. Violin plots are similar to box plots, except also present the probability density of the data. A boxplot is situated within the middle of the violin plot, with the area outside of the boxplot visually displaying the skewness of the biomarker distribution. We used ordinal logistic regression in order to compare biomarker concentration (log transformed and by quintiles) with the duration of AKI as an ordered categorical outcome. To assess the potential discrimination of the biomarkers, we calculated c-statistics for AKI duration for each biomarker alone and also in combination panels. When calculating the c-statistics using logistic regression models, two outcomes were used. AKI duration less than two days, including no AKI, was chosen to assess the ability of the biomarkers to distinguish individuals who experienced no or short duration AKI. The other outcome assessed was duration of AKI greater than or equal to 7 days, to determine the ability of the biomarkers to discriminate between those with and without long duration AKI. The best performing two and three-way biomarker combinations for both outcomes, defined as the highest c-statistics, were presented. We used multivariable Cox proportional hazards regression with AKI duration as the exposure and mortality as the outcome to assess the potential association of duration of AKI and long-term mortality. We considered a two tailed p value of <0.05 as significant.

Small cell counts (counts ≤ 5) are only presented for data collected by TRIBE-AKI and not from Ontario healthcare databases (where to minimize the risk of reidentification small cell counts are suppressed). All analyses were performed in SAS (version 9.3; SAS Institute, Cary, NC) and R 2.12.1 (R Foundation for Statistical Computing, Vienna, Austria) software.

## Results

### Patient Characteristics

Baseline characteristics between patients without AKI, and AKI by duration (1–2; 3–6 and ≥7 days) are presented in Table 1. Overall, 407/1199 patients (34%) experienced AKI. Of the 407 total with AKI, 251 (61.7%) had AKI lasting for 1–2 days; 118 (30%) had AKI for 3–6 days and 38 (8.3%) had AKI for ≥7 days. Of the 358 that had stage 1 AKI (Delta SCr ≥ 50% or ≥ 0.3 mg/dL), 115 (32.1%) had AKI duration of 3 days or more. In addition, 7 of 48 patients with stage 2 and 3 AKIN had short (1–2 days) duration of AKI. There was a graded relationship between increasing duration of AKI and diabetes, hypertension, congestive heart failure, and lower baseline estimated glomerular filtration rate compared with patients without AKI (P<0.01 for all). They were also more likely to undergo surgeries that had longer cross-clamp and to perfusion time and experience a complicated postoperative course, including more extra-renal complications, longer ventilator time, and ICU stays (P<0.001 for all).

**Table 1:** Patient Characteristics by Duration of AKI (n = 1194).

Characteristic	No AKI 0 days (n = 788)	1–2 days (n = 250)	3–6 days (n = 118)	≥ 7 days (n = 38)	P value
**Demographics**					
Age at the time of surgery, mean (SD)	71.1 (10.3)	71.8 (9.8)	71.1 (10.3)	73.3 (7.7)	0.49
Men	525 (67%)	176 (70%)	85 (72%)	29 (76%)	0.33
White race	740 (94%)	235 (94%)	112 (95%)	31 (82%)	0.02
**Medical History (prior to surgery)**					
Diabetes	294 (37%)	95 (38%)	58 (49%)	24 (63%)	<0.01
Hypertension	605 (77%)	205 (82%)	102 (86%)	31 (82%)	0.05
Congestive Heart Failure	175 (22%)	65 (26%)	49 (42%)	18 (47%)	<0.01
LVEF < 40%	77 (10%)	29 (12%)	11 (9%)	1 (3%)	0.37
Previous myocardial infarction	197 (25%)	61 (24%)	36 (31%)	10 (26%)	0.61
eGFR (mL/min per 1.73 m^2^), mean (SD)	69.2 (18.9)	66.4 (18.3)	61.3 (21.5)	57.2 (20.4)	<0.01
eGFR ≥ 60	548 (70%)	164 (66%)	56 (48%)	18 (47%)	<0.01
eGFR 30-<60	223 (28%)	79 (32%)	52 (44%)	18 (47%)	
eGFR < 30	17 (2%)	7 (3%)	10 (9%)	2 (5%)	
Pre-op Serum Creatinine§ (mg/dL), mean (SD)	1.05 (0.32)	1.10 (0.31)	1.23 (0.45)	1.30 (0.38)	<0.01
Urine albumin to Creatinine, mg/mmol					
< = 10.0	341 (43%)	83 (33%)	26 (22%)	6 (16%)	<0.01
10.0-<30	229 (29%)	74 (30%)	32 (27%)	12 (32%)	
30–300	188 (24%)	77 (31%)	43 (36%)	14 (37%)	
>300	30 (4%)	16 (6%)	17 (14%)	6 (16%)	
**Surgical Characteristics**					
Elective Surgery	656 (83%)	190 (76%)	78 (66%)	26 (68%)	<0.01
Surgery					
CABG	392 (50%)	121 (48%)	52 (44%)	12 (32%)	0.03
CABG & Valve	162 (21%)	56 (22%)	35 (30%)	16 (42%)	
Valve	234 (30%)	73 (29%)	31 (26%)	10 (26%)	
Off-pump	85 (11%)	20 (8%)	12 (10%)	6 (16%)	0.02
Re-do surgery	14 (2%)	4 (2%)	1 (1%)	1 (3%)	0.87
Perfusion time (minutes), mean (SD)	106.6 (52.7)	119.1 (59.6)	129.4 (74.2)	168.4 (91.3)	<0.01
Cross-clamp time (minutes), mean (SD)	72.7 (40.0)	80.7 (44.7)	90.9 (53.3)	114.4 (67.3)	<0.01
Number of Diseased Coronary Vessels					
None	219 (28%)	65 (26%)	26 (22%)	11 (29%)	0.78
One	106 (14%)	35 (14%)	13 (11%)	2 (5%)	
Two	149 (19%)	47 (19%)	22 (19%)	6 (16%)	
Three	314 (40%)	103 (41%)	57 (48%)	19 (50%)	
**Post-operative Complications**					
AKIN stage					
Stage 1	0	243 (97%)	95 (81%)	20 (53%)	
Stage 2	0	6 (2%)	14 (12%)	10 (26%)	
Stage 3	0	1(0%)	9 (8%)	8 (21%)	
Oliguria‡ in first day, n (%)	8 (1%)	3 (1%)	2 (2%)	2 (5%)	0.31
Delta Peak Serum creatinine (mg/dL), mean (SD)	0.03 (0.14)	0.37 (0.23)	0.75 (0.51)	0.98 (0.72)	<0.01
# Non-renal complications†, n (%)					
0	507 (64%)	152 (61%)	63 (53%)	6 (16%)	<0.01
1–2	232 (29%)	77 (31%)	37 (31%)	14 (37%)	
>2	49 (6%)	21 (8%)	18 (15%)	18 (47%)	
Ventilator > 48 hours, n (%)	8 (1%)	11 (4%)	10 (8%)	16 (42%)	<0.01
ICU LOS, mean (SD)	2.32 (4.11)	3.41 (7. 419)	3.64 (3.90)	20.32 (32.75)	<0.01
Hospital LOS, mean (SD)	6.89 (5.29)	9.32 (11.34)	9.18 (4.86)	32.05 (35.09)	<0.01

Data are presented as the mean (SD), n (%), or median (interquartile range) unless otherwise specified. eGFR, estimated GFR; SCr, serum creatinine.

^§^To convert serum creatinine values to millimoles per liter, multiply by 88.4.

^‡^Oliguria is defined as a patient who had 125 ml or 500 ml urine output in 6 or 24 hours, respectively.

^†^Nonrenal complications are defined as reoperation, infection, neurologic, pulmonary, vascular, and other.

### Biomarker Levels and Duration of AKI

**Fig 1:**
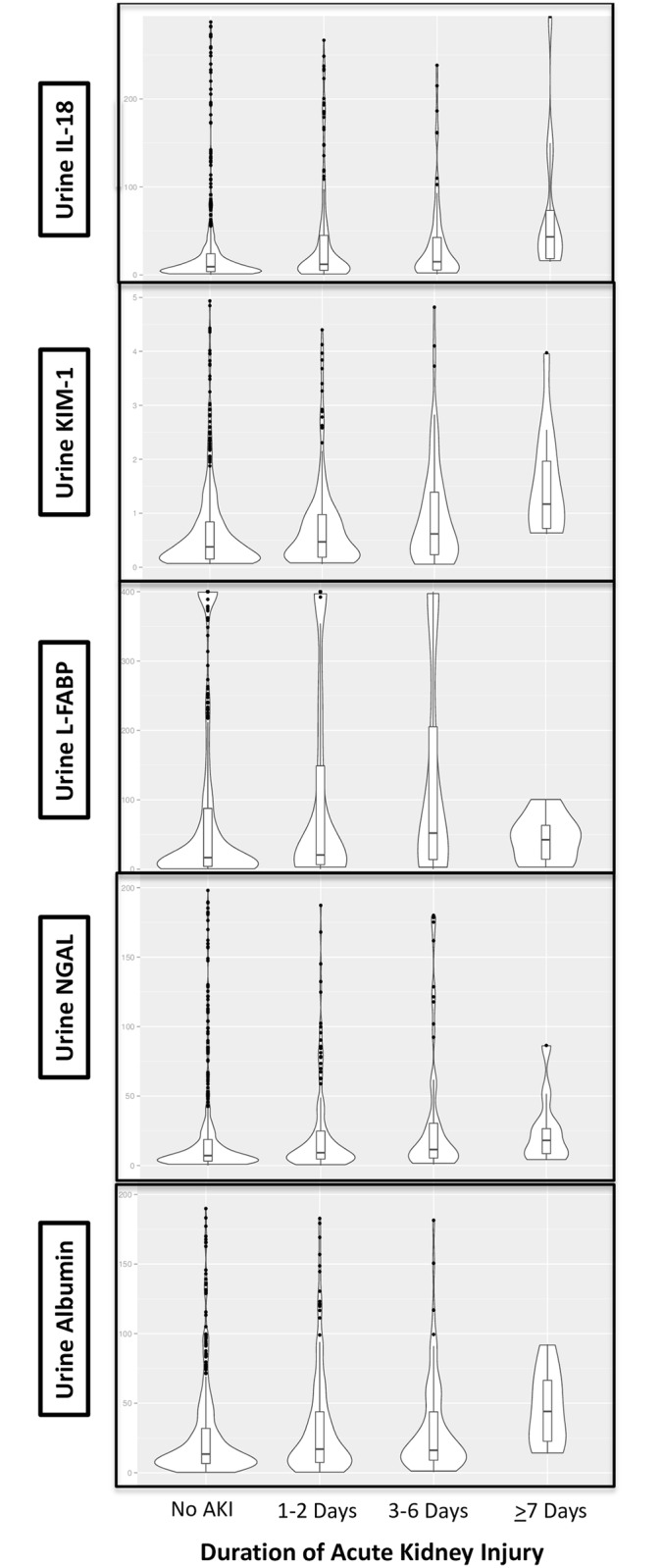
Violin Plots for Biomarker Levels With Acute Kidney Injury Duration. These violin plots demonstrate that patients with shorter duration of AKI tended to have a more skewed distribution of all 5 biomarkers, with their biomarker values skewed towards low values.

The median first postoperative values of all 5 kidney injury biomarkers were significantly higher as the duration of AKI increased (Fig 1). Shorter duration of AKI tended to have a more skewed distribution, with biomarker values skewed towards low values. In addition, the percentage of patients in highest quintile of biomarker concentrations increased as the duration of AKI increased, with the largest gradient observed for median IL-18 concentrations across AKI categories (Table 2).

**Table 2:** Median Values of Biomarkers by Duration of AKI and Odds Ratios per Log Increase in Biomarker for Increased Duration Category.

Biomarker 0–6 hours postop	No AKI (n = 781)	1–2 Days (n = 244)	3–6 Days (n = 116)	>7 Days (n = 36)	P Value	Unadjusted Odds Ratio (95% CI)	Adjusted Odds Ratio (95% CI)
Urine IL-18 (pg/mL)	9.3 (3.6–27.9)	15.82 (5.9–82.4)	23.1 (6.03–88.9)	45.9 (16.6–188.6)	<0.0001	1.29 (1.21–1.37)	1.22 (1.13, 1.32)
Urine NGAL (ng/mL)	8.4 (3.3–32.8)	14.1 (5.2–79.9)	15.0 (5.61–86.3)	26.5 (10.3–418.3)	<0.0001	1.16 (1.10–1.23)	1.06 (1.00, 1.14)
Urine KIM-1 (ng/mL)	0.37 (0.14–0.84)	0.53 (0.22–1.03)	0.71 (0.33–1.5)	1.02 (0.67–2.21)	<0.0001	1.44 (1.30–1.59)	1.36 (1.21, 1.52)
Urine L-FABP (ng/mL)	16.0 (3.3–78.9)	21.2 (6.4–178.7)	34.0 (6.6–183.3)	61.1 (13.9–371.9)	<0.0001	1.16 (1.10–1.23)	1.11 (1.04, 1.19)
Urine Albumin (mg/L)	12.5 (6.4–30.8)	18.0 (7.9–53.7)	20.0 (9.0–61.3)	43.3 (19.0–70.4)	<0.0001	1.28 (1.17–1.40)	1.20 (1.09, 1.32)

IQR = interquartile range (25^th^ percentile, 75^th^ percentile)

Adjustment variables were age, sex, white race, elective surgery, cardiopulmonary bypass time, preoperative eGFR, diabetes, hypertension, congestive heart failure, myocardial infarction, type of surgery, and site.

### Association of Postoperative Biomarkers and Duration of AKI

Higher levels of all 5 log transformed biomarkers were significantly associated with duration of AKI in univariable models, which persisted for all biomarkers except urine NGAL, after adjusting for confounders including age, sex, white race, elective surgery, cardiopulmonary bypass time, pre-operative eGFR, diabetes, hypertension, congestive heart failure, myocardial infarction, surgery type, and center. The strongest association were seen for urine KIM-1 and IL-18, where each log increase in these markers were associated with 36% and 22% increased odds of duration category, respectively (OR 1.36, 95% CI 1.21, 1.52 for KIM-1 and OR 1.22, 95% CI 1.13–1.32 for IL-18). The highest quintiles of KIM-1 and IL-18 were associated with 2.3-fold and 2.9-fold increased odds of duration of AKI, respectively (Table 3). In additional analyses using biomarkers normalized to urine creatinine, the associations were slightly attenuated but remained significant. (S1 Table)

**Table 3:** Associations by Quintiles of Biomarkers for Increased Duration Category.

First post-operative biomarker	Cut points	Unadjusted Odds Ratio (95% CI)	Adjusted Odds Ratio (95% CI)
**Urine IL-18 (pg/mL)**			
**Q1**	0.06–3.28	1.0 (Ref)	1.0 (Ref)
**Q2**	3.29–7.83	1.54 (1.02–2.34)	1.46 (0.93, 2.28)
**Q3**	7.84–16.93	1.70 (1.13–2.57)	1.46 (0.92, 2.32)
**Q4**	16.95–57.90	2.15 (1.43–3.22)	1.58 (0.99, 2.53)
**Q5**	58.22–11557.85	3.90 (2.62–5.78)	2.90 (1.80, 4.68)
**Urine NGAL (ng/mL)**			
**Q1**	0.05–3.13	1.0 (Ref)	1.0 (Ref)
**Q2**	3.15–6.93	1.65 (1.10–2.50)	1.31 (0.84, 2.03)
**Q3**	6.95–15.77	1.79 (1.19–2.69)	1.34 (0.86, 2.10)
**Q4**	15.88–96.31	2.61 (1.75–3.88)	1.67 (1.08, 2.59)
**Q5**	98.90–5486.26	2.67 (1.79–3.98)	1.52 (0.95, 2.42)
**Urine KIM-1 (ng/mL)**			
**Q1**	0.06–0.13	1.0 (Ref)	1.0 (Ref)
**Q2**	0.13–0.31	1.23 (0.81–1.86)	1.02 (0.66, 1.58)
**Q3**	0.32–0.61	1.53 (1.02–2.30)	1.35 (0.88, 2.08)
**Q4**	0.62–1.17	2.47 (1.67–3.66)	2.05 (1.34, 3.14)
**Q5**	1.17–26.69	2.96 (2.01–4.37)	2.30 (1.51, 3.53)
**Urine L-FABP (ng/mL)**			
**Q1**	0.06–2.86	1.0 (Ref)	1.0 (Ref)
**Q2**	2.91–10.59	1.61 (1.08–2.40)	1.57 (1.04, 2.38)
**Q3**	10.67–36.84	1.44 (0.96–2.16)	1.29 (0.84, 1.96)
**Q4**	37.15–162.06	1.86 (1.26–2.76)	1.77 (1.17, 2.67)
**Q5**	163.23–400.00	2.52 (1.71–3.71)	1.92 (1.26, 2.93)
**Urine Albumin (mg/L)**			
**Q1**	0.10–5.60	1.0 (Ref)	1.0 (Ref)
**Q2**	5.70–10.30	1.12 (0.75–1.68)	0.99 (0.65, 1.32)
**Q3**	10.40–20.80	1.34 (0.90–1.98)	1.17 (0.77, 1.76)
**Q4**	20.90–48.70	1.54 (1.04–2.27)	1.14 (0.76, 1.73)
**Q5**	48.80–2009.70	2.83 (1.94–4.12)	2.21 (1.48, 3.30)

Adjustment variables were age, sex, white race, elective surgery, cardiopulmonary bypass time, preoperative eGFR, diabetes, hypertension, congestive heart failure, myocardial infarction, type of surgery, and site.

### Diagnostic ability of Postoperative Biomarkers for Duration of AKI

The areas under the curve for AKI duration of ≤2 days (no AKI or AKI with duration up through 2 days) and AKI duration ≥ 7 days are presented in Table 4 for all postoperative biomarkers. The AUCs ranged from 0.62 (L-FABP) to 0.75 (KIM-1) for AKI duration ≥ 7 days. Panels of 2 or 3 biomarkers only improved the AUC to 0.75 and 0.76, respectively. The AUCs for AKI duration of ≤2 days ranged from 0.61 to 0.66, and improved to 0.68 with two or three biomarker panel combinations (Table 4).

**Table 4:** AUC using biomarker levels at 0–6 hours for the outcome of AKI duration.

	AKI Duration	
Biomarker Combination	≤2 Days	≥7 Days
**Individual biomarkers, day 1 (0-6h)**		
Urine IL-18	0.66 (0.03)	0.73 (0.04)
Urine NGAL	0.61 (0.03)	0.68 (0.04)
Urine KIM-1	0.65 (0.03)	0.75 (0.03)
Urine L-FABP	0.62 (0.03)	0.62 (0.05)
Urine albumin	0.63 (0.03)	0.72 (0.04)
**Best biomarker combinations**		
Two-way combinations		
Urine KIM-1 and urine IL-18	0.68 (0.03)	0.76 (0.03)
Three-way combinations		
Urine KIM-1, urine IL-18, and urine albumin	-	0.77 (0.03)
Urine KIM-1, urine IL-18, and urine NGAL	0.68 (0.03)	-

Two logistic regression models with binary indicator outcomes were used to obtain the AUCs; duration of 0–2 days and secondly, duration of greater than or equal to 7 days. Biomarker combinations with the highest c-statistic are presented as best biomarker combinations

### Duration of AKI and Long term Mortality

During the follow-up period (median 3 years, IQR (2.2, 3.6)), 12% of the entire cohort died. The crude mortality rates per 1000 person years increased from 42/1000 person-years for no AKI, 66/1000 person-years for 1–2 days of AKI, 87/1000 person-years for 3–6 days of AKI, and 254/1000 person-years for duration of AKI ≥7 days. The adjusted hazard ratios for mortality were roughly 1.5 (but borderline statistically significant) for duration of AKI of 1–2 and 3–6 days. Duration of AKI ≥7 days was associated with a four-fold increase in mortality risk compared to no AKI (Table 5).

**Table 5:** Association of Duration of Acute Kidney Injury with Mortality during Follow-up Period.

	Mortality Rate per 1000 person years	Unadjusted Hazard Ratio (95% Confidence Interval)	Adjusted Hazard Ratio (95% Confidence Interval)
No AKI (n = 788)	41.64	1.00 (Ref)	1.00 (Ref)
1–2 days (n = 250)	65.98	1.59 (1.08–2.35)	1.52 (1.00–2.31)
3–6 days (n = 118)	86.95	2.09 (1.36–3.22)	1.53 (0.95–2.45)
≥7 days (n = 38)	245.33	5.79 (3.87–8.68)	3.99 (2.75–5.79)

Mortality rates adjusted for center. Adjusted for age, sex, white race, elective surgery, pre-operative eGFR, diabetes, hypertension, congestive heart failure, myocardial infarction, surgery type, and center.

## Discussion

In this cardiac surgery cohort, elevated urinary levels of injury biomarkers, including IL-18, KIM-1, NGAL, L-FABP, and albumin, obtained immediately after surgery, were independently associated with duration of AKI in a graded manner. Moreover, while the vast majority of participants in the TRIBE-AKI Cohort experienced stage 1 AKI when classified by peak serum creatinine increase, approximately one-third had duration of AKI of at least 3 days or more. In contrast, as expected, the majority of patients with stage 2 or 3 AKI had duration of AKI that generally exceeded two days. Thus, the greatest incremental impact of application of urinary biomarkers to improve upon prediction of duration of AKI may be in those with more mild forms of AKI.

Consensus definitions of AKI account only for the magnitude of rise of serum creatinine,[9] which are based on a dose-response relationship between the severity of AKI (defined by serum creatinine) and outcomes.[10,11] While duration and magnitude of peak creatinine rise often are correlated, there are substantial number of individuals in which the two parameters can dissociate. Thus, the consensus definitions are limited by the fact that they do not consider duration of AKI as an input, as longer duration of AKI may help differentiate patients with prolonged intrinsic kidney injury from those who have transient AKI due to volume depletion or hemodynamic perturbations.[4] In addition, the duration of AKI may reflect the overall severity of illness, and thus the patients with continuing and severe illness will take longer to recover. In this cohort, it was readily apparent that patients with long duration of AKI generally had greater number of post-operative complications. Also, impediments to renal tubule repair and recovery, such as older age, less renal mass, or subclinical renal ischemia may impact duration of AKI.[12,13] Thus, the duration of AKI is a more multidimensional outcome incorporating etiology, severity and potential for recovery. Despite its potential as a reliable metric, AKI duration has not been used as an endpoint in clinical trials for intervention in or prevention of AKI.[14] However, a recent workshop convened by the National Institute of Diabetes and Digestive and Kidney Diseases considered utilizing duration as a secondary endpoint in AKI prevention after elective surgery. [15]

Indeed, if the goal is to enroll patients in a trial of an agent to treat post-operative AKI, enrolling patients with elevated biomarker levels of kidney injury immediately after surgery may identity a segment of patients with a high incidence of AKI, with a type of AKI that may be amenable to treatment. If a substantial proportion of participants with “short-duration of AKI” were enrolled, then it be would be challenging to demonstrate efficacy from any agent or strategy because recovery would be prompt regardless of the intervention.

Our study has important limitations. We did not have urine output data for building the definitions of AKI. Most of the AKI in the TRIBE-AKI cohort was stage 1. Thus, there is a small spectrum seen for the duration of AKI for those with stage 2 or 3 AKI based upon the peak creatinine rise.

In conclusion, in the TRIBE-AKI cohort, there was an independent dose-response association between urinary levels of injury biomarkers immediately after cardiac surgery and longer duration of AKI. Future studies should explore the potential utility of these urinary kidney injury biomarkers to enrich enrollment of patients at risk for longer duration of AKI into trials of interventions to prevent or treat post-operative AKI.

## Supporting Information

S1 Table Log-transformed and Quintile Associations of Urine Creatinine-Normalized Biomarkers for Increased Duration Category. Data presented as adjusted and adjusted odds ratios per log increase and by quintile of biomarker levels (with quintile 1 as reference). All biomarkers were normalized to urine creatinine. All estimates adjusted for age, sex, white race, elective surgery, cardiopulmonary bypass time, pre-operative eGFR, diabetes, hypertension, congestive heart failure, myocardial infarction, surgery type, and center.
(DOCX)
