# Supplementary material for: First Post-Operative Urinary Kidney Injury Biomarkers and Association with the Duration of AKI in the TRIBE-AKI Cohort
Source: PLoS One. 2016 Aug 18;11(8):e0161098. doi: 10.1371/journal.pone.0161098 (PMC4990204; doi:10.1371/journal.pone.0161098)
Supplement: S1 Table — Data presented as adjusted and adjusted odds ratios per log increase and by quintile of biomarker levels (with quintile 1 as reference). All biomarkers were normalized to urine creatinine. All estimates adjusted for age, sex, white race, elective surgery, cardiopulmonary bypass time, pre-operative eGFR, diabetes, hypertension, congestive heart failure, myocardial infarction, surgery type, and center. (DOCX) [file pone.0161098.s001.docx]

**S1 Table. Log-transformed and Quintile Associations of Urine Creatinine-Normalized Biomarkers for Increased Duration Category**

| **First post-operative biomarker** | | **Unadjusted Odds Ratio (95% CI)** | **Adjusted Odds Ratio (95% CI)** |
| --- | --- | --- | --- |
| **Urine IL-18 (pg/mL)** | **log** | 1.30 (1.20, 1.39) | 1.25 (1.15, 1.35) |
|  | **Q1** | 1.0 (Ref) | 1.0 (Ref) |
|  | **Q2** | 1.13 (0.73, 1.75) | 1.05 (0.67, 1.65) |
|  | **Q3** | 2.10 (1.40, 3.15) | 1.81 (1.18, 2.78) |
|  | **Q4** | 2.63 (1.76, 3.93) | 2.34 (1.52, 3.59) |
|  | **Q5** | 3.62 (2.44, 5.39) | 2.95 (1.90, 4.57) |
| **Urine NGAL (ng/mL)** | **log** | 1.12 (1.06, 1.19) | 1.06 (1.00, 1.14) |
|  | **Q1** | 1.0 (Ref) | 1.0 (Ref) |
|  | **Q2** | 1.46 (0.98, 2.18) | 1.22 (0.80, 1.86) |
|  | **Q3** | 1.64 (1.10, 2.45) | 1.40 (0.92, 2.12) |
|  | **Q4** | 2.15 (1.46, 3.18) | 1.66 (1.09, 2.52) |
|  | **Q5** | 2.02 (1.37, 2.99) | 1.45 (0.94, 2.23) |
| **Urine KIM-1 (ng/mL)** | **log** | 1.60 (1.38, 1.86) | 1.57 (1.34, 1.83) |
|  | **Q1** | 1.0 (Ref) | 1.0 (Ref) |
|  | **Q2** | 1.31 (0.88, 1.97) | 1.39 (0.91, 2.11) |
|  | **Q3** | 1.47 (0.99, 2.19) | 1.58 (1.04, 2.39) |
|  | **Q4** | 1.62 (1.09, 2.41) | 1.59 (1.05, 2.41) |
|  | **Q5** | 2.94 (2.01, 4.31) | 2.81 (1.88, 4.20) |
| **Urine L-FABP (ng/mL)** | **log** | 1.10 (1.04, 1.16) | 1.10 (1.04, 1.17) |
|  | **Q1** | 1.0 (Ref) | 1.0 (Ref) |
|  | **Q2** | 1.34 (0.91, 1.98) | 1.26 (0.84, 1.89) |
|  | **Q3** | 1.18 (0.80, 1.76) | 1.17 (0.77, 1.77) |
|  | **Q4** | 1.76 (1.20, 2.58) | 1.88 (1.25, 2.81) |
|  | **Q5** | 1.79 (1.22, 2.62) | 1.76 (1.17, 2.66) |
| **Urine Albumin (mg/L)** | **log** | 1.18 (1.07, 1.30) | 1.17 (1.06, 1.30) |
|  | **Q1** | 1.0 (Ref) | 1.0 (Ref) |
|  | **Q2** | 1.33 (0.90, 1.97) | 1.11 (0.74, 1.67) |
|  | **Q3** | 1.06 (0.71, 1.59) | 0.99 (0.65, 1.51) |
|  | **Q4** | 1.73 (1.18, 2.54) | 1.64 (1.10, 2.45) |
|  | **Q5** | 2.04 (1.39, 2.98) | 1.90 (1.28, 2.84) |
